# The role of Extracellular Genomic Materials (EGMs) in psychiatric disorders

**DOI:** 10.1038/s41398-023-02549-5

**Published:** 2023-07-18

**Authors:** Ayşe Kurtulmuş, Cemal Çağıl Koçana, Selin Fulya Toprak, Selçuk Sözer

**Affiliations:** 1 Department of Genetics, Aziz Sancar Institute of Experimental Medicine, Istanbul University, Istanbul, Turkey; 2 Institute of Health Sciences, Istanbul University, Istanbul, Turkey; 3 Istanbul Göztepe Prof.Dr.Süleyman Yalçın City Hospital, Department of Psychiatry, Istanbul, Turkey

**Keywords:** Personalized medicine, Clinical genetics

## Abstract

Extracellular Genomic Materials (EGMs) are the nucleic acids secreted or released from all types of cells by endogenous or exogenous stimuli through varying mechanisms into the extracellular region and inevitably to all biological fluids. EGMs could be found as free, protein-bound, and/ or with vesicles. EGMs can potentially have immunophenotypic and/or genotypic characteristics of a cell of origin, travel to distant organs, and interact with the new microenvironment. To achieve all, EGMs might bi-directionally transit through varying membranes, including the blood–brain barrier. Such ability provides the transfer of any information related to the pathophysiological changes in psychiatric disorders in the brain to the other distant organ systems or vice versa. In this article, many aspects of EGMs have been elegantly reviewed, including their potential in diagnosis as biomarkers, application in treatment modalities, and functional effects in the pathophysiology of psychiatric disorders. The psychiatric disorders were studied under subgroups of Schizophrenia spectrum disorders, bipolar disorder, depressive disorders, and an autism spectrum disorders. EGMs provide a robust and promising tool in clinics for prognosis and diagnosis. The successful application of EGMs into treatment modalities might further provide encouraging outcomes for researchers and clinicians in psychiatric disorders.

## Introduction

Psychiatric disorders are a group of complex diseases affecting mood, thinking, perception and behavior. They rank among the most disabling disorders with prevalence estimates and disability weights comparatively higher than many other diseases [1]. World Health Organization (WHO) reported in 2019, that psychiatric disorders are the second leading cause of disabilities, with no evidence of a global reduction in the burden since 1990 [2, 3]. Prevalence figures indicate that up to 35–40% of people have a psychiatric disorder at some point during their lifetime [4]. Unfortunately, the health systems worldwide cannot fully respond to the treatment requirements of psychiatric disorders for the time being.

Despite their massive burden on individuals, families, and society, there is still no curative treatments for these disorders. Treatment options are a few and limited with the most studies barely indicating moderate response rates. The lack of definitive information regarding the cause and pathophysiology, coupled with the limited availability of biological-based guidance for clinicians, poses a significant challenge to the development of more effective treatment modalities. Uncovering pathophysiological fingerprints of psychiatric disorders is lagging behind due to the involvement of complex multifactorial mechanisms, heterogeneous presentation of disorders and difficulties in classification of patients. Therefore, further research is essential for ushering to shed light on the underlying mechanisms and to explore and develop molecular characteristics of psychiatric disorders with the aim of providing more accurate diagnosis and achieving early and personalized treatment.

Extracellular Genomic Materials (EGMs) are the general nomenclature of all extracellular molecules of nucleic acids found in a variety of biological fluids, including blood, cerebrospinal fluid (CSF), amniotic fluid, lymphatic circulation, saliva, urine, milk, etc. [5, 6]. The term “EGMs” has been first used by our research group recently to cover various types of freely circulating cell-free genomic materials [7]. In recent years, it has been revealed that EGMs deliver essential information in various pathologies including cancer, and provide valuable advancements in terms of diagnostic, prognostic and theranostic approaches. Despite the remarkable breakthroughs in other disciplines of medicine, the potential use of EGMs is less appreciated in psychiatric research. In fact, considering their bi-directional transit capability through the blood–brain barrier (BBB) and their ability to transfer information of their cell of origin to target cells, EGMs might have a great value for diagnosis, prevention and treatment of psychiatric disorders [8].

This narrative review intends to evaluate and spotlight the potential roles of EGMs' in psychiatric disorders. In this regard, we first described the concept of EGMs and then reviewed the relevant literature for psychiatric disorders. Finally, we discussed the potential applications of EGMs in the clinical aspects of psychiatry.

## Extracellular Genomic Materials (EGMs)

Nucleic acids including DNA, RNA, and related molecules, are released by all cell types into the extracellular environment during both physiological and pathological processes and subsequently pass through the borders [9]. Central nervous system (CNS) cells such as neurons, microglia, astrocytes and oligodendrocytes, as well as many other cell types can release EGMs during their cellular functions or as a result of apoptosis or necrosis [10–12].

**Fig. 1:**
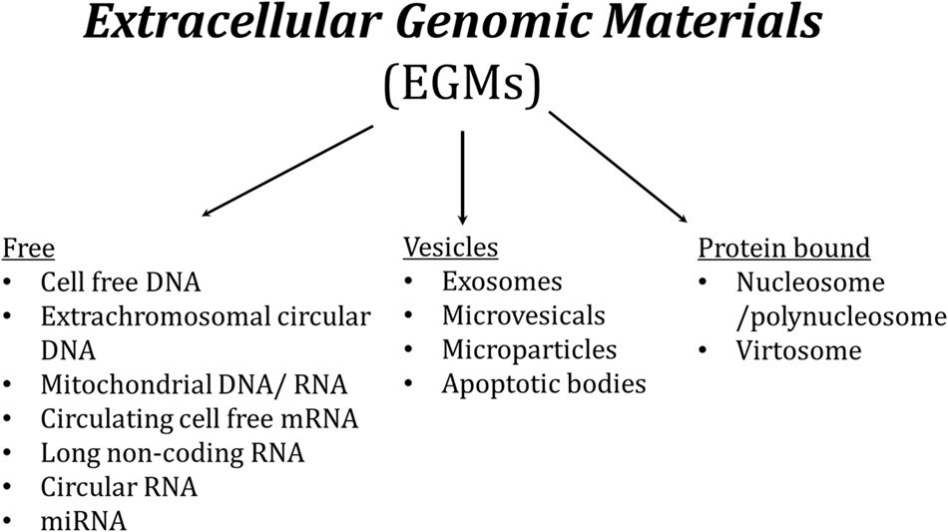
Classification of extracellular genomic materials (EGMs). Subgroups of EGMs could be studied in free, vesicular or protein bound form.

The nucleic acids in the extracellular environment differ by their genetic materials, nucleic acid structures, the complexes they form with other molecules, and the assemblies in which they are involved. In this context, EGMs can be divided into three main groups including free, vesicular, or protein-bound (Fig. 1). Free EGMs circulate nakedly and might consist of but not limited to cell-free DNA (cfDNA), extrachromosomal circular DNA (cirDNA), cell-free mitochondrial DNA (cf-mtDNA) /RNA (cf-mtRNA), circulating cell-free mRNA, long noncoding RNA, circular RNA, and miRNA etc. [13]. EGMs can also exist in protein or lipid-bound forms in the extracellular space, forming complex structures such as, nucleosome/polynucleosomes and virtosomes [14, 15]. Vesicular EGMs are enclosed within extracellular vesicles (EVs) with varying sizes and names, such as exosomes (30–100 nm), microparticles (100–1000 nm), microvesicles (50–1000 nm), or apoptotic bodies (50–5000 nm) with some other molecules [16]. The EVs released from CNS cells might contain DNAs, RNAs, proteins, and metabolites of the cell of origin as their cargo. These EVs play a pivotal role in intercellular communication and facilitate the transmission of their cargo to recipient/target cells. By acting as message carriers both within the CNS and between the brain and periphery, EGMs mediate a cascade of downstream reactions in numerous physiological and pathological processes, triggering signal transduction and regulating functional state of target cells. Thus, this ability provide EGMs with wide range of essential roles in neurogenesis, myelination, synaptic plasticity, neuronal survival and neuroinflammation [17]. In the context of diseases, EVs released from cells may carry diverse cargoes that differ from their healthy counterparts [18].

**Fig. 2:**
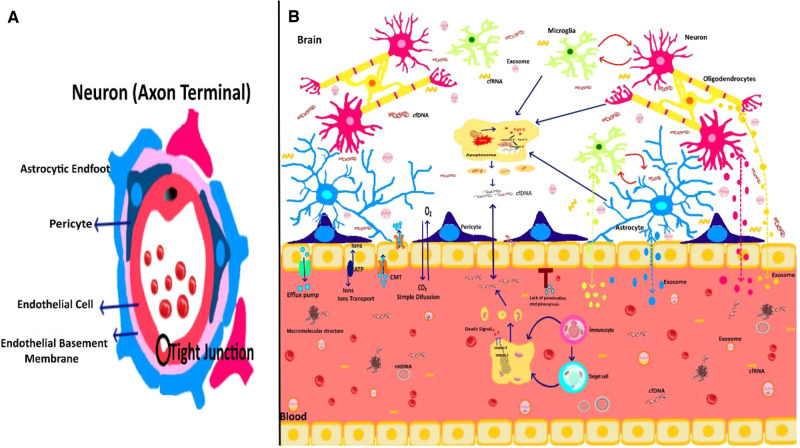
The Function of EGMs in Central Nervous System. **A** The structure of the blood–brain barrier (BBB) is shown. On the surface of the brain capillaries, endothelial cell come together with tight junctions to form a monolayer. Endothelial cells act as a physical and metabolic barrier. The basement membrane (BM), pericytes, and astrocytes contribute directly or indirectly to the BBB as a barrier. **B** EGMs are involved in dynamic interactions between circulation and neuronal network. The transport system of BBB includes ion transport, efflux pump, simple diffusion, carrier-mediated transport (CMT) and pinocytosis. In and out transport of EGMs between circulation and CNS occurs via BBB.

The release mechanism of EGMs, whether active or passive, depends on the type of EGMs being delivered. For instance, cfDNA tends to be passively released, whereas EVs exhibit a preference for active release [5, 19]. The blood–brain barrier (BBB), which is a semi-permeable endothelial cell-based border between CNS and circulatory system, is a highly selective barrier. It regulates brain hemostasis by working as both physical and metabolic barrier, which diligently transports nutrients and small hydrophobic molecules and also protects CNS by preventing transfer of immune factors, antibodies, and immune cells from the blood [20–22]. The interaction between blood vessels and brain cells, known as "neurovascular unit", is involved in regulating BBB function. A simplified representation of the neurovascular unit is shown in Fig. 2. Studies have demonstrated that EGMs, including EVs, can cross the BBB in both direction with the assistance of endothelial cells [23]. The hydrophobic surface of EV’s lipid bilayer plays a crucial role in activating various mechanisms in endothelial cells, including endocytosis, phagocytosis, micropinocytosis, physical contact, or plasma fusion, allowing EVs to cross BBB [24]. The ability of EGMs to cross the BBB enhances their functional roles in distant organ systems. In the CNS, both endogenous and exogenous EVs can be detected simultaneously. Exogenous EVs enter the CNS through the BBB from the peripheral circulation [25], endogenous EVs originate from the brain and transfer into the peripheral circulation [26]. Under normal conditions, the transport of EVs is tightly regulated, but certain pathological conditions may increase the permeability of the BBB, facilitating their transport [27–29]. The cargo materials carried by EVs may also play a role in determining how they cross the BBB. Additionally, EVs participate in waste removal processes and mediate elimination of neurotoxic aggregates/proteins within the CNS [17].

EGMs exhibit characteristics that are reflective of the cells from which they originate and provide profound information about the specific cell or tissue of origin [5, 30, 31]. The genetic and epigenetic profiles of EGMs such as distinct mutations on nucleic acids and specific methylation patterns, as well as immunophenotypic properties such as cell type-specific markers on EVs may provide further insight into the cell of origin, and allow a deeper understanding of the cellular characteristics and functions involved in disease mechanisms [10, 32]. It is important to note that, EGMs have a broader role beyond intercellular communication. Depending on their specific genetic material, they can initiate various mechanisms in target cells. A recognizable example would be from tumor cells that might release EGMs with a mutation [10] or oncogenes [33]. When these EGMs target healthy cells, they can impact their physiology and trigger pathophysiological processes, potentially leading to the development of diseases [7, 34–36]. Since, they can be easily detected from the periphery and have specific biosignatures of the cell of origin, EGMs hold great potential as noninvasive biomarkers for screening, diagnosis and treatment of psychiatric disorders and might provide a window into the brain cells and their microenvironment.

In light of these knowledge, there are four common key features of EGMs which make them indispensable for psychiatric research:

1. EGMs possess the capability to cross blood–brain barrier in both directions.
2. EGMs carry specific immune/genetics/epigenetics signatures that reflect the cells/tissues from which they have originated.
3. EGMs play an important role in intercellular communication within the CNS and between the CNS and other systems.
4. EGMs can be easily isolated from the peripheral sources in a relatively noninvasive manner.

Investigating the quantity and composition of EGMs holds significant potential in various applications. The concentration of EGMs in circulation is dynamic and can be influenced by a range of physiological and pathological processes. Factors such as exercise, stress, pregnancy, inflammation, sepsis, cancerogenesis, and transplantation and CNS-related disorders can increase EGM levels [37–41]. Conversely, certain diseases or some other temporary conditions, as well as medical treatments, can lead to a decrease in EGMs [42–44]. Apoptosis of CNS cells can also boost EGM levels [45]. cfDNA has a short half-life in circulation (15 min to 1 h) [46], whereas EVs are better preserved in circulation and travel to such distant regions [38]. Since there are several types of EGMs with different characteristics, it is crucial to understand the underlying mechanisms and identify and evaluate all factors that significantly influence the EGMs production from various sources. In certain diseases, studying cfDNA may be essential, while in others, extracellular RNA or EVs may be more informative, providing valuable insights for researchers and clinicians [47]. Therefore, it is essential to consider the eligibility and characteristics of EGMs for their employment in various applications such as diagnosis, monitoring and treatment modalities.

## EGMs and psychiatric disorders

### Free EGMs

#### Cell-free DNA

CfDNAs depending on its origin might include genomic (cfDNA), mitochondrial (cf-mtDNA) and ribosomal (cf-rDNA) DNA all of which are detectable nucleic acid fragments that are either released from CNS cells through varying essential physiological processes or apoptosis or necrosis of the cells. Studies indicated that up to 80% of CNS cells induce apoptosis which has been proven to be involved in the pathophysiological processes of various psychiatric disorders [45]. CfDNA is also a member of damage-associated molecular patterns (DMAPs) that is recognized by pattern recognition receptors (PRRs) and promotes inflammation. Therefore, cfDNA might be of particular interest for psychiatric disorders, given the abnormal immune response observed in patients [48]. Not only quantitative levels of cfDNA but also their genetic markers and methylation patterns and fragmentation profiles can be valuable for investigating disease mechanisms.

In this section, we summarized cfDNA, cf-rDNA, and cf-mtDNA studies in psychiatric disorders (Table 1).

**Table 1:** Application of Extracellular Genomic Materials in Psychiatric Diseases.

*Condition/Disorder*	*EGM*	*Source*	*Sample size*	*Method*	*Findings*	*Reference*
Schizophrenia spectrum disorder	*cfDNA*	Blood	58 male SZ 11 male schizophreniform 14 male Alcohol-induced PD 30 male HCs	*cfDNA index* *Fluorescence* *İmmunoassay*	• Increased level of 8-oxodG- cfDNA in SZ • No difference in cfDNA index between study groups	[49]
		Plasma	100 SZ 60 HCs	*Fluorometric quantification* *Flow cytometry*	• Higher cfDNA in SZ • Increased 8-oxodG, NOX-4 and BCL-2 levels in SZ	[50]
		Plasma	65 SZ 29 mood disorders 62 HCs	*Fluorometric quantification* Spectroscopy qPCR	• Twofold higher and shorter cfDNA levels in SZ • No difference in cfDNA level in mood disorders	[51]
		Serum	164 SZ 48 MDD 29 Alcohol-induced PD	Rt-PCR ELISA	• Alu repeat sequence increase in SZ • Alu repeat associated with IL-1β and IL-8 levels	[53, 54]
		Serum	174 SZ 100 HCs	qRT-PCR	• A significant increase in cfDNA levels in SZ • A progressive decrease in cfDNA levels with antipsychotic treatment in a subgroup of 57 newly diagnosed patients	[54]
		Plasma	29 FEP 31 HCs	Multiplex PCR NGS	• Higher total, neuron, astrocyte, oligodendrocyte, and whole brain-derived cfDNA levels in patients	[55]
	*Cf-rDNA*	Blood	100 male SZ 96 male HCs	Nonradioactive Quantitative Hybridization	Increased level of cfDNA and cf-rDNA in SZ	[52]
	*Cf- mtDNA*	Plasma	34 first-episode SZ 28 HCs	Rt-PCR	No difference in Cf-mtDNA level between groups	[56]
	*Nucleosome*	Serum	30 SZ 30 HCs	ELISA	Increased level of nucleosome in SZ	[88]
	*EV (circular RNA)*	Plasma	11 SZ 11 HCs	qRT- PCR	38 upregulated and 6 downregulated circRNAs in SZ that have potential roles in the pathogenesis of disease	[98]
	*EVs* *miRNA*	Blood	49 first-episode SZ 46 HCs	qRT- PCR	• Increased level of hsa-miR-206, which regulates BDNF expression, in SZ	[91]
		Plasma	138 FEP 134 HCs	qPCR	Increased exosomal miR-137 and decreased COX6A2 levels in FEP which were associated with worse positive and negative symptoms, cognitive impairment and functioning.	[94]
		Blood	14 drug-naïve FEP 10 HCs	Nanosight technology Electron microscopy qRT-PCR	• Increased miR-203a-3p levels and decreased DJ-1 mRNA and protein in FEP • Olanzapine returned DJ-1 and miR-203a-3p levels to the levels seen in HCs	[131]
		Plasma	9 treatment-resistant SZ 9 HCs Replication set: 50 non-treatment-resistant SZ 50 HCs	Microarray qPCR	• Upregulated miR-675-3p in treatment-resistant SZ compared to HCs • A decreased level of miR-675-3p in non-treatment-resistant schizophrenia cohort compared to HCs	[132]
	*EVs long noncoding RNA (lncRNA)*	Blood	104 SZ (56 drug free, 48 under risperidone treatment) 96 HCs	qRT- PCR	Increased expression of MIAT and PVT1 in serum exosomes of SZ	[99]
*Bipolar disorder*	*cfDNA*	Serum	20 drug-free BD 20 HCs	Singleplex real-time PCR	Higher level of cf-nuclear DNA and lower levels of cf-mtDNA in BD	[57]
	*Cf-mtDNA*	Serum	64 BD (adolescant) 41 HCs	qRT- PCR	No difference between groups for cf-mtDNA	[59]
		Plasma	10 BD (during both depression and remission phase of the same patients) 10 HCs	Multiplex immunoassay qRT- PCR	No significant difference in cf-mtDNA copy number between groups No correlation between plasma cf-mtDNA levels and plasma IL-6 levels	[60]
	*EVs (miRNA)*	Plasma	69 BD (15 depressed, 27 manic, 27 euthymic) 41 HCs	qPCR	• Increased levels of miR-484, -652-3p, -142-3p and decreased level of miR-185-5p in BD • Findings did not differ according to the disease stage	[101]
		Plasma	20 BD-1 21 HCs	Microarray	33 nominally significant microRNAs altered in BD patients	[102]
*Major depression disorders*	*cf-mtDNA*	Plasma	55 MDD 50 HCs	qRT-PCR	Increased level of cf-mtDNA levels in MDD	[62]
		Plasma	32 late-life depression 21 HCs	qRT-PCR	• Higher cf-mtDNA levels in patients • A significant correlation between ccf-mtDNA levels and the severity of depression	[63]
		Plasma	281 MDD 49 HCs	qRT-PCR	• Lower cf-mtDNA in both patients with current and remitted depression compared to controls. • No significant difference in ccf-mtDNA between current and remitted depression • treatment with mood stabilizers lamotrigine, valproic acid or lithium was associated with lower cf-mtDNA	[58]
		Plasma	109 MDD 28 BD 17 SCZ 29 HCs	qRT-PCR Multiplex immunoassay	• Lower plasma mtDNA copy number in MDD compared to other groups • A correlation between mtdNA copy number and IL-4 levels in MDD	[65]
		Serum	21 female MDD 27 HCs	real-time-PCR	• No difference between groups in terms of cf-mtDNA levels • No correlation between cf-mtDNA levels and severity of depression	[66]
	*EVs (miRNA)*	Plasma	4 treatment-resistant depression 4 HCs	NGS	Upregulated hsa-miR-335-5p an downregulated hsa-miR-1292-3p in patients	[106]
		Blood	33 drug-naïve MDD 46 HCs	qRT-PCR	Increased hsa-miR-139-5p expression level in patients	[103]
		Serum	52 MDD 31 HCs	qRT-PCR	Higher expression levels of miR-146a in MDD compared to HCs • let-7e, miR-145, and miR-146a showed acceptable discrimination between the remission and non-remission groups	[105]
		Serum	30 MDD 30 HCs	qRT-PCR	Increased exosomal miR-139-5p levels in MDD patients compared to controls	[104]
		Serum	Discovery set: 9 drug-free MDD (adolescants) 8 HCs Validation set: 34 drug-free MDD (adolescants) 38 HCs	RT-PCR	Increased miR-450a-2-3p, miR-556-3p, and miR-2115-3p levels in patients	[108]
		Serum	6 MDD 3 HCs	RT-PCR	Increased expression of expression of miR-9-5p in patients	[107]
	*EVs (NDEVs)*	Plasma	40 MDD (20 treatment-responder, 20 nonresponder) 20 HCs	miRNA sequencing Western blot qpCR	NDEVs from depressed patients were smaller than controls and NDEV size increased with treatment response. Changes in miR-21-5p, miR-30d-5p and miR-486-5p in NDEV were associated with treatment response	[109]
*Autism spectrum disorders*	*cfDNA*	Plasma	96 ASD 34 HCs	Flourimetric Analysis Flow cytometry	Higher plasma cfDNA levels in autism patients • cfDNA of especially severe ASD patients contained a high amount of oxidative modification	[133]
		Serum	20 ASD 12 HCs	*qRT-PCR*	• amount of mtDNA significantly higher for mt-CytB and for mt-7S in ASD	[68]
	*EVs*	Serum	20 ASD 8 HCs	Transmission electron microscopy (TEM) ELISA	• Extracellular vesicles of ASD do not differ in size or shape • Amount of total EV-related protein is higher in ASD	[110]
	*EVs (lncRNA)*	Plasma	14 ASD 14 HCs	*qRT-PCR* Nanoparticle tracking analysis (NTA) Transmission electron microscopy (TEM)	• SVAT-lncRNAs of *SLC18A2*, *SYT9*, *STX8*, and *SYT15* were measured to be upregulated • SVAT-lncRNAs of *SV2C* and *SYP* were minimally expressed	[111]
	*EVs (lncRNA)*	Serum	100 children with ASD 60 HCs	L1 (L1CAM)-captured EVs (LCEVs) lncRNA microarray RNA-sequencing	A total of 1418 mRNAs, 1745 lncRNAs, and 11 miRNAs were differentially expressed, and most of them were downregulated in ASD. • The levels of EDNRA, SLC17A6, HTR3A, OSTC, TMEM165, PC-5p-139289_26, and hsa-miR-193a-5p were validated in at least 15 ASD and 15 TD individual serum samples	[112]
*Other psychiatric disorders*	*EVs (miRNA)*	Plasma	10 MDP 10 HDP 10 HCs	*qRT-PCR* *Nanoparticle tracking analysis (NTA)* *Transmission electron microscopy (TEM)*	• no differences in both exosome size distribution and numbers between controls and patients • 34 differentially expressed miRNAs between HCs and MDPs (7 upregulated, 27 downregulated) • 19 differentially expressed miRNAs between HCs and HDPs (7 upregulated, 12 downregulated) • No difference in miRNAs expression between MDPs and HDPs	[113]
		Plasma	12 PTSD 12 HCs Validation cohort 10 PTSD 10 HCs	NGS *qRT-PCR*	• EV and EVD fractions were different between groups • Expression of 11 miRNAs and 6 piRNAs were different in PTSD	[114]
		Serum	48 PTSD 47 HCs	*qRT-PCR*	• PTSD group displayed higher expression of composite marker 1 (miR-200b-3p, miR-433-3p, miR-10a-5p, miR-10b-5p, miR-199a-3p, miR-224-5p, miR-146a-5p, and miR-143-3p) and composite marker 2 (miR-1247-5p, miR-363-5p, miR-346-5p, and miR-486-5p) as compared with control group	[115]
	*Cf-mtDNA*	Plasma	46 SAD 42 HCs	*qRT-PCR*	• SAD patients had significantly lower cf-mtDNA • Ni significant change in cf-mtDNA levels after CBT	[69]

*SZ* Schizophrenia, *HCs* Healthy controls, *MDD* Major depressive disorder, *FEP* First-episode psychosis, *BD* Bipolar disorder, *ASD* Autism spectrum disorder, *PTSD* Post-traumatic stress disorder, *SAD* Social anxiety disorder, *MDP* Metamphertamine-dependent patients, *HDP* Heroin-dependent patients.

##### Schizophrenia spectrum disorders

The first study investigating cfDNA levels in schizophrenia spectrum disorders (SSD) was published in 2017. The study included 83 male patients with acute psychotic disorders (58 schizophrenias, 11 schizophreniform disorder, and 14 alcohol-induced psychotic disorder) and 30 male healthy control subjects. It explored the role of oxidative DNA damage and dysregulation of apoptosis in the disease pathophysiology by evaluating the concentration of plasma cfDNA and the levels of 8-oxodG in cfDNA and lymphocytes. They found no difference in the cfDNA index between study groups. However, patients had elevated 8-oxodG levels in both cfDNA and lymphocytes. Moreover, the cfDNA/ FL1-8-oxodG ratio, an indicator of apoptosis in damaged cells, was elevated in one-third of patients with schizophrenia [49]. Ershova et al. showed increased cfDNA concentrations in patients compared to healthy controls with higher cfDNA concentrations being associated with higher 8-oxodG levels in lymphocytes [50]. Another study investigating size distributions and concentrations of cfDNA in schizophrenia showed approximately twofold higher cfDNA levels in patients with schizophrenia. Furthermore, the cfDNA molecules in these patients displayed a distribution pattern resembling apoptosis, with shorter DNA molecules being more prevalent. In the same study, there was no difference between healthy controls and patients with mood disorders in cfDNA levels [51]. In Ershova et al. study, mean plasma cfDNA concentration was twofold higher in untreated male schizophrenia subjects compared to healthy controls. The authors also investigated cell-free ribosomal DNA (cf-rDNA) concentration, blood leukocyte DNA, and cfDNA rDNA copy numbers. They found that cf-rDNA concentration was ~threefold higher in the patient group compared to controls and that both leukocyte DNA and cfDNA rDNA were higher in patients. Authors hypothesized that the elevated levels of cf-rDNA, which contains many unmethylated CpG motifs, may potentially activate the TLR9-MyD88-NF-κB signaling pathway and production of proinflammatory cytokines and, therefore, can be related to abnormal cytokine levels reported in patients with schizophrenia [52]. Qi et al. explored cfDNA Alu repeat in schizophrenia, major depressive disorder, and alcohol-induced psychotic disorder. They found an increased Alu concentration in Schizophrenia compared to the other two study groups, which were also found effective in differentially diagnose between schizophrenia and other disorders. Moreover, Alu concentrations were positively correlated with IL-1β levels in all groups and IL-18 levels in patients with schizophrenia, in particular [53]. Another study investigating cfDNA levels in schizophrenia revealed that cfDNA levels reliably distinguish patients from controls and found a significant increase in cfDNA levels in the patient group before treatment, which decreased progressively after treatment with antipsychotics [54]. In a recent study, researchers investigated the presence of brain-specific cfDNA in patients with first-episode psychosis. They employed DNA methylation markers specific to the brain, and found significantly elevated neuron, astrocyte, oligodendrocyte, and whole brain-derived cfDNA levels [55]. Ouyang et al. investigated cf-mtDNA levels in first-episode schizophrenia patients. They found no significant changes between patients and controls regarding cf-mtDNA copy number. However, a significant decrease was observed in the patient group by antipsychotic treatment, and the CN changes were significantly correlated with changes in symptom severity [56].

##### Bipolar disorders

cfDNA studies in bipolar disorder mainly consist of studies focusing on circulating cf-mtDNA. The only study investigating cf-nuclear DNA levels in bipolar disorder showed that cf-nuclear DNA levels were higher in the patient group compared to the controls. However, there was no difference between groups regarding cf-mtDNA [57, 58]. Another study comparing cf-mtDNA levels in the adolescent group reported that patients did not differ from controls with regards to ccf-mtDNA levels [59]. Kageyama et al. compared the cf-mtDNA levels of 10 patients with bipolar-1 disorder in both depressive and remission phases with ten healthy controls. They reported that there was no significant difference observed between the groups. In addition, no correlation was found between cf-mtDNA levels and IL-6 [60]. Ho et al. compared rapid-cycling and nonrapid-cycling bipolar patients. They showed that cfDNA methylation patterns differed between the groups, and this difference was particularly evident in the gene regions associated with CNS, synapses, and glutamate transmission [61].

##### Depressive disorders

There are a few studies investigating cf-mtDNA levels in major depressive disorder. Lindqvist et al. found that patients with major depressive disorder had higher cf-mtDNA levels in comparison to controls, and there was a positive correlation between cf-mtDNA and antioxidant glutathione peroxidase levels in the whole sample. Among a subgroup of patients who underwent an eight-week treatment with selective serotonin reuptake inhibitors, contrasting changes in cf-mtDNA levels were observed between treatment non-responders and responders. Non-responders exhibited an increase in cf-mtDNA levels, while cf-mtDNA levels in the responders remained unchanged [62]. In a separate study investigating cf-mtDNA levels in late-life depression, it was found that patients had higher cf-mtDNA levels. Furthermore, the levels of cf-mtDNA were correlated with the severity of depression but not with cognitive functions [63]. However, Ampo et al. investigated cf-mtDNA levels in older adults with late-life depression and frailty and found no significant difference between patients with late-life depression without frailty and healthy controls [64]. Another study examining cf-mtDNA levels in depressed patients reported lower levels of cf-mtDNA in patients than the controls, but no significant difference in cf-mtDNA levels was observed between patients with current depression and those with remitted depression. In the same study, the use of mood stabilizers was found to be associated with lower cf-mtDNA levels [58]. A study comparing plasma mtDNA copy numbers in major depressive disorder, bipolar disorder, schizophrenia, and control groups found that mtDNA copy number (CN) was lower in the major depressive disorder compared to all other study groups and mtDNA CN was significantly correlated with IL-4 levels in major depressive disorder patients. There was no difference between schizophrenia and control groups regarding mtDNA copy number. At the same time, mtDNA CNs of bipolar disorder patients were found to be lower compared to controls [65]. Contrarily, Behnke et al. found no difference between depressed female patients and healthy controls regarding cf-mtDNA levels [66].

##### Autism spectrum disorder

Only one study investigates cfDNA concentrations in Autism spectrum disorder. Shmarina et al. showed that patients with Autism spectrum disorder had elevated plasma cfDNA levels compared to controls and contained a high amount of oxidative modification. It was shown that the expression of the NFкB1, nuclear factor gene and levels of proinflammatory cytokines were also higher in the peripheral lymphocytes of the patients, and it was hypothesized that oxidized cfDNA fragments lead to the activation of inflammatory processes [67]. Another study on ASD indicated that the serum of young autistic children contained the significantly higher levels of mtDNA for mt-CytoB and mt-7S [68].

##### Other psychiatric disorders

In a recent study, Lindquvist et al. showed a decreased cf-mtDNA level in patients with social anxiety disorder in comparison to healthy controls. Cf-mtDNA level did not change in the patient group after CBT, and was not associated with the severity of symptoms [69].

#### Circulating cell-free miRNAs

MicroRNAs (miRNA) are small noncoding RNAs that consist of 19–23 nucleotides. They exert functional effects on cells by negatively regulating gene expression and/or potentially interacting with cell receptors as ligands. They can inactivate a target mRNA by binding its 3′- UTR side and diminish their post-transcriptional output. Approximately 70% of known miRNAs are expressed in the CNS, contributing to various biological processes including neurogenesis, neuroplasticity, neuronal differentiation, function and maintenance, and overall brain development. The ability of miRNAs to regulate gene function, coupled with their abundant expression levels in CNS suggest their potential role as epigenetic modifiers in the pathophysiology of psychiatric disorders [70]. miRNAs can be detected not only in tissue also in all bodily fluids. Circulating miRNAs (cimiRNA) are produced in the nucleus and can be actively or passively released into the circulatory system. The active release of cimiRNAs usually occurs via EVs or in free form [71]. As cimiRNAs can easily be collected, they have been of particular interest as biomarkers for clinical applications. Table 2 represents a summary of circulating cell-free miRNA studies in psychiatric disorders. In line with the scope of this review, we only focused on the studies with serum and plasma miRNA in this section and excluded whole blood and PBMC. In addition, EV miRNA studies have been summarized in the relevant section below.

**Table 2:** Circulating cell-free miRNA levels in psychiatric disorders.

	*Method*	*Source*	*Sample size*	*Upregulated*	*Downregulated*	*Reference*
Schizophrenia spectrum disorder	*Solexa Sequencing and TLDA assay* *qRT-PCR*	Plasma	164 SZ 187 HCs (Test cohort) 400 SZ 213 HCs 162 non-schizophrenia psychiatric disorders (Validation Cohort)	miR-130b miR-193a-3p		[72]
	*qRT-PCR*	Plasma	50 Z 50 HCs	miR-7		[134]
	*qRT-PCR*	Serum	115 SZ 40 HCs	miR-181b miR-219-2-3p miR-1308 let-7g miR-346 miR-92a	miR-195 miR-17	[74]
	*qRT-PCR*	Plasma	25 SZ 13 HCs	miR-132 miR-195 miR-30e miR-7		[135]
	*qRT-PCR*	Plasma	20 SZ 20 HCs	miR-181b miR-30e miR-34a miR-7		[73]
	*qRT-PCR*	Plasma	61 SZ 62 HCs	miR-181b miR-30e miR-346 miR-34a miR-7		[75]
	*qRT-PCR*	Plasma	215 SZ (104 early onset, 111 adult onset) 100 HCs 72 unaffected first-degree relatives	miR-137 miR-34b miR-34c		[76]
*Bipolar disorder*	*qRT-PCR*	Plasma	21 BD-1, manic 21 HCs		miR-134	[77]
	*qRT-PCR*	Plasma	66, BD, euthymic (under lithium treatment) 66 HCs	miR -132 miR -134 miR -152 miR -607 miR -633 miR -652	miR-15b miR-155	[70]
	*qRT-PCR*	Serum	79 BD-2 95 HCs (Training group) 20 BD-2 20 HCs (Testing group)	miR-7-5p miR-23b-3p miR-142-3p miR-221-5p miR-370-3p		[136]
	*NanoString nCounter system miRNA assay* *qRT-PCR*	Plasma	15 BD (drug free, manic) 9 HCs	miR-150-5p miR-25-3p miR-451a miR-144-3p (miR-25-3p and miR-144-3p validate by PCR)	miR-363-3p miR-4454 + miR-7975 miR-873-3p miR-548al miR-598-3p miR-4443 miR-551a miR-6721-5p (miR-6721-5p validated by PCR)	[137]
	*NGS* *qRT-PCR*	Plasma	18 BD 50 MDD 37 HCs (discovery cohort) 26 BD 84 MDD 74 HCs (validation cohort)	let-7e-5p miR-125a-5p (both in BD and MDD patients)		[138]
*Depressive disorders*	*qRT-PCR*	Plasma	35 MDD 35 HCs (test cohort) 100 MDD 100 HC 50 BD 50 SCZ		miR-134	[81]
	*qRT-PCR*	Plasma	50 MDD 41 HCs	miR-451a miR-17-5p miR-223-3p	miR-320a	[139]
	*Microarray* *qRT-PCR*	Plasma	16 MDD 14 HC	miR-320d miR-101-3p miR-106a-5p miR-423-5p miR-93-5p		[140]
	*qRT-PCR*	Plasma	159 patients with depression, anxiety, stress or adjustment disorder 52 HCs		miR-144-5p	[141]
	*qPCR*	Serum	18 drug-free MDD 17 HCs	miR-124-3p		[142]
	*qRT-PCR*	Serum	40 MDD 40 HCs	miR-132 miR-182		[79]
	*qRT-PCR*	Serum	6 MDD 6 HCs	miR-221-3p miR-34a-5p let-7d-3p	miR-451a	[143]
	*NGS* *qRT-PCR*	Plasma	63 late-life depression 53 HC		miR-1-3p miR-184 (only the latter one was validated)	[144]
	*qRT-PCR*	Plasma	45 MDD, drug-free 32 MDD treated with citalopram 32 HCs	miR-132 miR-124		[145]
	*qRT-PCR*	Serum	39 MDD 36 HCs		miR-16 miR-135a miR-1202	[146]
*Autism spectrum disorders*	*qRT-PCR*	Plasma	110 ASD 113 HCs	miR-191-5p miR-151a-3p miR-139-5p miR-181a-5p miR-432-5p miR-181b-5p miR-195-5p miR-328-3p miR-106a-5p miR-484		[147]
	*NGS*	Plasma	60 ASD (35 severe, 25 mild) 8 HCs	miR-302b-3p miR-302a-3p miR-302d-3p miR-144-5p miR-106a-5p miR-144-3p miR-122-5p miR-4306 miR-885-3p miR-182-5p miR-183-5p	miR-584-5p miR-99a-5p miR-4433b-5p miR-127-3p miR-12136 miR-6852-5p miR-744-5p miR-134-5p miR-23a-5p miR-221-3p miR-4433b-3p	[84]
	*qRT-PCR*	Serum	159 ASD 159 HCs	miR-19b-3p	miR-181b-5p miR-320a	[148]
	*qRT-PCR*	Serum	38 ASD 28 HCs	miRNA-197-5p miRNA-664a-3p miR-424-5p	miR-500a-5p	[149]
	*qRT-PCR*	Serum	45 ASD 21 HCs 33 unaffected siblings and 74 parents		miR-19a-3p miR-361-5p miR-3613-3p miR-150-5p miR-126-3p miR-499a-5p	[85]
	*qRT-PCR*	Serum	30 ASD 30 HCS		miR-3135a miR-328-3p	[150]
	*microRNA array* *RT-PCR assay*	Serum	30 ASD 25 HCs	miR-140-3p		[151]
	*stemloop qRT-PCR pooling assay*	Serum	30 ASD 30 HCS	miR-365a- 3p miR-619-5p miR-664a-3p	miR-3135a miR-328-3p miR-197-5p miR-500a-5p miR-424-5p	[152]
	*miRNA PCR Array* *qPCR*	Serum	55 ASD 55 HCs	miR-101-3p miR-106b-5p miR-130a-3p miR-195-5p miR-19b-3p	miR-151a-3p miR-181b-5p miR-320a miR-328 miR-433 miR-489 miR-572 miR-663a	[153]
	*miRNA microarray* *qRT-PCR*		2 ASD 3 HCs 18 ASD 20 HCs	miR-557 miR-486-3p		[154]
*Other psychiatric disorders*	*qRT-PCR*	Plasma	30 OCD 30 HCs	miR-132 miR-134		[155]
	*NGS*	Serum	8 PTSD 6 HCs	miR-218-2-3p miR-3609 miR-432-5p miR-138-5p miR-221-5p miR-4485-3p miR-31-5p miR-146b-5p miR-5096 miR-222-3p miR-1273g-3p miR-302a-5p miR-221-3p miR-619-5p miR-335-5p miR-146b-3p miR-3175 miR-3656	miR-184 let-7d-5p miR-98-5p miR-146a-5p	[156]
	*qRT-PCR*	Plasma	57 HDP 46 HCs	miR-320a let-7b-5p		[157]
	*microRNA array* *RT-PCR assay*	Serum	42 HDP 42 MDP 42 HCs	let-7b-5p miR-206 miR-486-5p (in heroin abusers) miR-9-3p (in methamphetamine abusers)		[158]
	*microRNA array* *RT-PCR assay*	Plasma	25 IGD 26 HCs (discovery cohort) 20 IGD 16 HCs		miR-26b-5p miR-200c-3p miR-652-3p	[159]

*SZ* Schizophrenia, *HCs* Healthy controls, *BD* Bipolar affective disorder, *MDD* Major depressive disorder, *ASD* Autism Spectrum disorder, *OCD* Obsessive-Compulsive disorder, *PTSD* Post-traumatic stress disorder, *HDP* Heroin-dependent patients, *MDP* Metamphetamine-dependent patients, *IGD* Internet gaming disorder, *TLDA* TaqMan low-density array, *qRT-PCR* quantitative reverse transcription polymerase chain reaction, *NGS* next generation sequencing, *miRNA* microRNA.

##### Schizophrenia spectrum disorders

Mounting evidence suggests that miRNAs have the potential to serve as biomarkers for early and precise diagnosis of schizophrenia [72]. There are studies indicating an increase in miR-181b-5p, miR-7, miR-30e, miR-34a and miR-346 levels in the serum and plasma of patients with schizophrenia [73, 74]. Song et al. found that miRNA-181b, miRNA-30e, miRNA-34a and miRNA-7 were upregulated in drug-free schizophrenia patients compared to controls and the expression level of miRNA-181b significantly decreased following treatment which was also correlated with an improvement of negative symptoms [73]. Sun et al. showed that the expression level of miR-181b, miR-30e, miR-346, miR-34a and miR-7 were upregulated in drug-free patients with schizophrenia and levels of miR-181b, miR-132, miR-30e and miR-432 significantly decreased after 6 weeks of treatment. The expression changes in miR-181b, miR-132, miR-30e and miR-212 were correlated with symptom improvement [75]. In another study, it was demonstrated that miR-130b and miR-193a-3p were upregulated in patients with schizophrenia, but not in those with non-schizophrenia psychiatric disorders, when compared to healthy controls. Additionally, the levels of these miRNAs decreased in patients who remitted after a one-year follow-up period with an atypical antipsychotic drug, while no such decrease was observed in patients who did not achieve remission [72]. The expression levels of miR-137, miR-34b, and miR-34c have been reported to be upregulated not only in patients with schizophrenia but also their unaffected first-degree relatives [76].

##### Bipolar disorders

There are several studies in the literature investigating cimiRNAs as a biomarker for bipolar disorders (Table 2). Various downregulated and upregulated miRNAs have been reported in patients with bipolar disorders, however, neither of them has been established as a reliable biomarker, so far. Rong et al. showed a decreased level of plasma miR-134 in drug-free patients compared to healthy controls. miR-134 levels increased after 4 weeks of treatment, however the difference between controls and patients remained significant even after treatment [77]. Tekdemir et al. [70] found that miR-652, miR-633, miR-607, miR-152, miR-134 and miR-132 levels were significantly upregulated in the plasma of euthymic bipolar patients, while the miR-15b and miR-155 levels were significantly downregulated. Notably, the level of miR-155-5p was found to be associated with the severity of disease [70].

##### Depressive disorders

Studies conducted on cimiRNAs have revealed that miR-124-3p, miR-132-3p, miR-182-5p, miR 345-5p, and miR-425-3p are frequently upregulated and miR-636 is downregulated in patients with major depressive disorder [78] (Table 2). In a study performed by Li et al., it was found that the expression of *BDNF* gene, which plays a crucial role in the pathophysiology of disorder, was decreased in patients with major depressive disorder, while the levels of miR-182-5p and miR-132-3p were increased [79]. Similarly, Su et al. was observed the increased level of miR-132-3p and decreased level of BDNF in the patients’ blood [80]. Zhang et al. found that level of plasma miR-134 was significantly downregulated in patients with major depressive disorder, which significantly increased after 8-weeks of treatment in those with treatment response but still remained lower than healthy controls. They also indicated that miR-134 level was significantly decreased in major depressive disorder compared to patients with bipolar disorder and Schizophrenia spectrum disorders [81]. Li et al. showed that lower levels of miR-199b-5p, miR-143-3p, miR-200a-3p, and miR-215-5p were associated with future risk of relapse in people with history of recurrent major depressive disorder [82]. Another prognostic study also indicated that baseline plasma level of let-7b-5p was negatively associated with higher risk of developing major depressive disorder later on [83].

##### Autism spectrum disorders

More consistent results have been reported for cimiRNA changes in patients with ASD. Most studies indicated upregulated levels of miR-19b-3p, miR-106a-5p, miR-195-5p and miR-664a-3p, whereas downregulated levels of miR-181b-5p, miR-3135a, miR-320a, miR-328-3p and miR-500a-5p in ASD (Table 2). Salloum-Asfar et al., revealed that hsa-miR-291a-3p (hsa-miR-302a-3p, hsa-miR-302b-3p, hsa-miR-302d-3p) family was upregulated in patients with ASD compared to healthy controls. They also showed that miR-302 family and miR-135b-5p were expressed at higher levels in patients with severe symptoms than those with mild symptoms [84]. Ozkul et al., indicated decreased levels of serum miR-19a-3p, miR-126-3p, miR-150-5p, miR-361-5p, miR-3613-3p, and miR-499a-5p in patients with ASD. Similarly, unaffected relatives of patients also exhibited a decrease in these miRNAs, albeit to a lesser extent [85].

### Protein-bound EGMs

During the process of apoptosis or necrosis, the cell undergoes fragmentation and degradation. This leads to the release of various residues from the cellular genome, such as fragmented chromosomes, DNA, RNA, DNA/RNA-related molecules, and mitochondrial genome. These components are released into the circulation in the form of nucleosomes and polynucleosomes. A nucleosome is comprised of a segment of DNA that is wrapped around a core of eight histone proteins [86]. These molecular complexes play a crucial role in protecting nucleic acids from degradation by extracellular nucleases, effectively stabilizing them for a certain period. Additionally, as part of the cellular functions, living cells have the ability to release newly synthesized DNA and RNA through lipoproteins known as virtosomes. These molecular complexes have the capability to incorporate their cargo DNA or RNA into the genome of the target cells, potentially resulting in some changes in the biology of target cells [15, 87]. So far studies about virtosomes are limited and none in psychiatric disorders. However, there is one study that focuses on nucleosomes and examines the impact of oxidative stress on nucleosome levels, as well as its relationship with the clinical features of patients with schizophrenia. In the study, the serums of thirty patients with schizophrenia and 30 healthy controls were studied and found higher nucleosome levels in patients with schizophrenia [88] (Table 1).

### EGMs with extracellular vesicles

EVs are nanoparticles that are secreted from almost all kind of cells and play a significant role in various biological and pathological processes. The three main types of EVs including exosomes, microvesicles and apoptotic bodies are well characterized depending on their sizes and the molecules carried on the surface of EVs [89]. We, herein, used the generic term ‘EV’ while summarizing the relevant literature, with the aim of avoiding incorrect definitions and confusion. EVs hold a great potential as circulating and noninvasive biomarkers of complex neuropsychiatric disorders and have attracted more attention in psychiatric research in recent years [90].

EVs are stable and abundant in circulation and can be isolated from peripheral blood and cerebrospinal fluids. The markers on EVs provide valuable information about the origin of EV and the content might be influenced in CNS pathologies. EVs have an important role for the communication between the brain and other organs/systems. Even that our primary focus is the genomic materials in EVs in psychiatric disorders, we also briefly summarized findings of protein and metabolite cargoes in a separate section below as they are carried together within the cargo of EVs and it is not possible to disregard their effect in mediating cell function/ signal transduction.

#### RNAs in extracellular vesicles

RNAs encapsulated within EVs mainly include lncRNAs, mRNAs, miRNAs and circRNAs. Among them special attention has been given to miRNAs.

##### Schizophrenia spectrum disorders

Du et al. [91] performed genome-wide miRNA expression profiling on exosomes derived from drug-naïve first-episode patients. They identified 11 miRNAs that could be utilized to classify patients and controls with a high accuracy rate of up to 90%. They also validated the sequence data using qRT-PCR in a larger sample. They confirmed that the sequence data consistently showed an increased exosome level of hsa-miR-206, which regulates BDNF expression, in patients with schizophrenia [92, 93]. In their preclinical and clinical phase study, Khadimallah et al. [94] showed increased plasma exosome miR-137 and decreased *COX6A2* levels in first-episode psychosis as well as some alterations in mitophagy markers that can lead to accumulation of damaged mitochondria and exacerbate oxidative stress and parvalbumin interneurons impairment. Indeed, patients with high exosome miR-137 and low *COX6A2* levels exhibited more impaired auditory steady-state response associated with worse cognitive impairment, global and social functioning, and more severe positive and negative symptoms than those with miR-137 and *COX6A2* levels in the range of controls [94]. In a study by Tsoporis et al., blood-derived exosomes from drug-naïve first-episode schizophrenia patients were examined. The study revealed decreased levels of DJ-1 mRNA and protein, a redox-sensitive protein that has a neuroprotective effect in the brain. Additionally, increased levels of miR-203a-3p, which can target the 3′ UTR of DJ-1, were observed in patients. Six weeks of olanzapine monotherapy restored DJ-1 antioxidant levels through the regulation of miR-203a-3p expression [95]. Funahashi et al. investigated global expression changes of miRNAs in plasma exosomes of treatment-resistant schizophrenia patients by using microarray analysis and identified 13 upregulated and 18 downregulated miRNAs that were relevant to neuronal and brain development. Among them, the upregulated expression of miR-675-3p was validated through qPCR. Furthermore, the expression level of miR-675-3p was significantly decreased in the non-treatment-resistant schizophrenia cohort compared to healthy controls [96]. In a post-mortem study, miRNA expression analysis first examined exosomal miRNA levels obtained from the prefrontal cortices of eight patients with schizophrenia, nine patients with bipolar disorders, and twelve healthy control subjects. The findings were then validated with RT- PCR. In another study, it has been shown that the expression of miR- 497 in schizophrenia and miR-29c in patients with bipolar disorders is increased compared to healthy controls [97].

Tan et al. explored alterations in plasma exosome circular RNA (circRNAs) expressions in patients with schizophrenia. 44 circRNAs exhibited differential expression pattern in patients compared to controls with 38 upregulated and six downregulated circRNAs. Further validation was performed on eight circRNAs with high fold change using qRT-PCR in a separate sample (*n*:6 for each group), and four circRNAs were confirmed to show positive results. Bioinformatics analysis of the identified circRNAs revealed their potential involvement in pathways relevant to pathophysiology of diseases, including metabolic processes, stress response, and histone ubiquitination [98]. Guo et al. investigated expression levels of 15 serum exosome lncRNAs in patients with schizophrenia. They found a significant difference in the expression of MIAT and PVT1 between drug-free patients and healthy controls with MIAT levels having good discriminatory performance. However, patients who used risperidone for 1-month did not differ from controls in terms of MIAT and PVT1 levels, while there was a significant difference between drug-free and treatment groups [99]. Apart from these studies conducted on schizophrenia spectrum disorders, in a recent study by Tomita et al. identified six urinary exosome miRNAs expressed differently in persistent psychotic-like experiences. This study prompts that urine exosome miRNAs might be used as novel biomarkers [100].

##### Bipolar disorders

Ceylan et al. examined circulating exosome miRNA expressions in bipolar disorder (*n* = 69; 15 depressives, 27 manic, and 27 euthymic patients) and showed that miR-142-3p, -484 and -652-3p were consistently upregulated while miR-185-5p was downregulated in patients, irrespectively of the disease state. Bioinformatics analysis highlighted some pathways for those dysregulated miRNAs, such as PI3K/Akt signaling pathway, fatty acid metabolism, extracellular matrix, and adhesion pathways [101]. In another study with a small sample size, miRNA levels in peripheral blood extracellular vesicles of bipolar-1 patients were examined, and it was shown that the expression of 33 miRNAs varied in the patient group. Some of these miRNAs were compatible with miRNAs reported in previous post-mortem studies and identified to play a role in some brain-related pathways [102].

##### Depressive disorders

Wei et al. performed a genome-wide miRNA expression profiling on blood-derived exosomes from major depressive disorder patients and determined that hsa-miR-139-5p was the top differentially expressed exosome miRNA with increasing levels in patients and showed a good performance in distinguishing groups from each other. It was also shown that injection of exosomes isolated from patients with major depressive disorder into normal mice revealed depression-like behaviors in mice. An alleviation of depressive-like behaviors was observed when exosomes isolated from healthy subjects were administered to stress-treated mice [103]. These results validated in another study showing increased level of serum exosome miR-139-5p in major depressive disorder patients [104]. Hung et al. also examined miRNA levels in serum exosomes from patients with major depressive disorder and found that miR-146a levels were higher in patients than in controls. Patients who achieved remission after antidepressant treatment had lower miR-145, miR-146a, let-7e, miR-21-5p and miR-155 before treatment compared to those who did not, and the levels of these miRNAs increased after treatment in the remission group. The levels of let-7e, miR-145, and miR-146a successfully distinguished remission and no remission groups [105]. In a tiny cohort of four patients with treatment-resistant depression and four healthy controls, it has been shown that hsa-miR-335-5p was upregulated and hsa-miR-1292-3p was downregulated in plasma exosomes isolated from treatment-resistant depression patients [106]. In the clinical phase of their study, Xian et al. showed that the expression of miR-9-5p was increased in serum-derived exosomes of patients with major depressive disorder which can promote polarization of M1 microglia and lead to neuronal injury by over releasing proinflammatory cytokines [107]. In adolescents with major depressive disorder, miR-556-3p, miR-450a-2-3p and miR-2115-3p levels have been found increased in serum EVs isolated from patients and among them miR-450a-2-3p was found to mediate the association between childhood trauma and the development of major depressive disorder in adolescent [108]. Saeedi et al. [109] investigated neuron-derived extracellular vesicles (NDEV) and their cargo in relation to antidepressant response in patients with major depressive disorder. They found that NDEVs from patients with depression were smaller than healthy controls, however, an increase was observed in the size of NDEVs with antidepressant treatment response. Furthermore, changes in the cargo of NDEVs, particularly in miR-486-5p, miR-21-5p and miR-30d-5p were associated with treatment response. Additionally, the targets of these miRNAs were found to be altered in anterior cingulate cortex of individuals who committed suicide [109].

##### Autism spectrum disorders

In children with ASD, Tsilioni et al. showed that EVs isolated from patients did not differ in size or shape in comparison to healthy controls, whereas the amount of total EV-associated protein and the amount of mtDNA contained in EVs were higher in the patients. It has also been shown that EVs isolated from patients lead to higher expression of IL-1β in human microglia cultures compared to those isolated from controls [110]. Fang et al. investigated the dynamic profiles of differentially expressed synaptic vesicle-associated transcripts (SVATs) in circulating plasma exosomes of children with ASD. SVAT-lncRNAs of *SYT15*, *STX8*, *SYT9* and *SLC18A2* were upregulated, and SVAT-lncRNAs of *SYP* and *SV2C* were downregulated in patients compared to healthy children. SVAT-mRNAs at the *SYP, SYT9* and *SYT15* loci were upregulated, whereas SVAT-mRNA at the *SV2C* locus was downregulated [111]. Qin et al. performed whole transcriptome analyses of L1CAM-captured EVs in children with ASD and revealed that the majority of differentially expressed RNAs in patients were involved in networks related to neurons and glycans [112].

##### Other psychiatric disorders

Besides major psychiatric disorders discussed above, a few studies investigated exosome content in other psychiatric conditions. Chen et al. investigated circulating exosomes from heroin-dependent patients (HDPs) and methamphetamine-dependent patients (MDPs). They found no difference in exosome size distribution or numbers between controls and patient with substance use disorders. However, 34 and 19 miRNAs were identified as differentially expressed in MDPs and HDPS compared to HCs. There was no significant difference in miRNA expression levels of HDPs and MDPs [113]. Lee et al. performed comprehensive profiling of miRNAs in different components of blood, including whole plasma, EVs and EV depleted plasma, derived from combat veterans with PTSD and control subjects. They showed that miRNA profiles in each fraction differed. 11 miRNAs and six piRNAs showed PTSD-associated changes in EVs, among which increased miR-203a-3p was validated in an independent cohort. miR-203a-3p is associated with serotonergic, dopaminergic, and cholinergic synapses, various signaling pathways, metabolism, inflammation, and cell cycle [114]. Kang et al., also revealed that a set of circulating exosome miRNAs showed altered expression in subjects with PTSD and associated with symptom severity. The authors suggest that those miRNAs have the potential to serve as epigenetic markers of PTSD [115].

#### Protein and metabolite cargo

Ranganathan et al. examined the plasma exosome levels of various proteins that are more specific to CNS cells and are known to play a role in neurodevelopment in a sample of 24 patients with schizophrenias and 12 controls and showed that GFAP concentrations were higher and a-II-Spectrin levels were lower in the patient group. No difference was found between the groups in synaptophysin levels. There was no difference in the mean exosome concentrations or sizes of the groups [116]. In another metabolomics study, it has been shown that the levels of 25 metabolites (10 of which are increased, 15 are decreased) in plasma exosomes of patients with schizophrenia differ from controls and that these metabolites have an accuracy of 82–99% in distinguishing patients from controls. Bioinformatics analyses have shown that these metabolites are effective in pathways such as glycerophospholipid metabolism, which may contribute to the pathophysiology of schizophrenia. Again no difference was found in the size and number of exosomes between the groups in this study [117]. In addition, there are studies in the literature that specifically examine the cargo contents of exosomes originating from CNS cells. In a recent study, electron transport chain and complement protein levels of astrocytes and neuron-derived exosomes obtained from the plasmas of 10 first-episode psychosis patients and ten healthy controls were examined, and some electron transport chain proteins (subunits 1 and 6 of NADH-ubiquinone oxidoreductase (complex I) and the subunit 10 of cytochrome b-c1 oxidase (complex III)) concentrations were found to be decreased, in contrast, to complement mediator levels (C3b opsonin, C5b-9 attack complex). There was no difference between the groups in the amount and size of exosomes [118]. Again by the same group, a significant decrease in mitochondrial functional protein levels such as ATP synthase, mitofusin 2, and cyclophilin in astrocyte and neuron-derived exosomes in patients with first-episode psychosis in astrocyte-derived extracellular vesicles and NDEVs [119] and altered levels of mitochondrial calcium ion channel proteins such as an increased level of CACNA- 1C and decreased levels of LETM1, NCLX, SLC24A6, and TRPM4 in plasma NDEVs of patients with first-episode psychosis [120] have been reported. In another study, it was shown that Aβ42 levels in astrocyte-derived exosomes (ACE) were higher in patients with schizophrenia compared to controls, and high exosome ACE-P-T181-tau levels in the whole sample were associated with impaired executive functions [121]. Kapogiannis et al. [122] also showed changes in protein levels that play a role in the insulin signaling pathway in neuronal-derived exosomes of drug-naïve first-episode psychosis errors and obtained findings supporting the role of insulin metabolism disorders (neuronal insulin resistance) in the etiopathology of the disease, independent of antipsychotic treatment [122].

Du et al. conducted a study analyzing metabolites in serum exosomes of patients with bipolar disorder. They findings revealed that 15 specific metabolites (Lysope 18:0, Lysope 14:0, Chenodeoxycholic Acid, 13-oxoODE, N-Acetylmethionine, 1-Naphthylacetic Acid, Glycine, D-2-Aminobutyric Acid, 2-Aminoethanesulfonic Acid, Phosphoric Acid, Lysopc 18:0, Lysopc 20:1, Glucosamine, Biopterin, and PAF C-16) demonstrated good to excellent performance in differantiating bipolar disorders from both healthy controls and other major mental disorders. Bioinformatics analysis revealed that these metabolites are related to sugar metabolism, which suggests that dysfunction in sugar metabolism may potentially contribute to the onset and/or development of bipolar disorder [123]. In an in vitro study, iPSCs generated from somatic cells (fibroblasts) of bipolar patients and healthy controls were differentiated into astrocytes, and then exosomes released from astrocytes were examined. It was reported that exosomes from both groups were of similar size but that exosomes from patients were somewhat more concentrated [124].

In a study with patients with major depressive disorder it was found that BDNF levels were low and proBDNF levels were high in the serum and exosomes of patients compared to controls, and BDNF levels increased, and proBDNF levels decreased with treatment [125]. Nasca et al. showed that the number of CNS-derived exosomes increased in the plasmas of patients, and the insulin receptor substrate-1 levels in its content were lower compared to the controls, and increased insulin receptor substrate-1 levels in patients were associated with anhedonia and suicidality [126]. In Jiang et al. study, 20 differentially expressed exosome proteins were identified by the label-free quantitative proteomics between major depressive disorder and healthy control subjects. Among them, plasma component proteins were predominantly found to be elevated in exosomes derived from patients. Notably, SERPINF1, a glycoprotein known for its neuroprotective properties, exhibited the most significant downregulation in the same group [127].

## Clinical implications and future directions

The etiology of psychiatric disorders has not been fully elucidated, and most of these disorders still lack curative treatments. Current treatment modalities only offer a certain level of improvement in response, highlighting the pressing need for studies on the neurobiological mechanisms of psychiatric disorders. Moreover, the diverse clinical presentations of the same diseases, substantial variability in treatment response among patients, and the potential involvement of distinct etiological biological processes even within patients sharing the same diagnosis highlight the importance of personalized and individualized treatment options and the search for specific biomarkers. The emergence of personalized medicine and the integration of various molecular techniques such as genomics, transcriptomics, and epigenomics into clinical practice have revolutionized the diagnosis, prognosis and therapeutic monitoring of diseases. EGMs have garnered significant attention in medicine in recent years, as they hold the potential to serve as biomarkers for diagnosis or companion diagnostics and be utilized as tools in treatment approaches. This paper aimed to provide a comprehensive review of EGM studies, which represent a relatively novel area of interest in psychiatric research.

One of the primary advantages of integrating EGMs into clinical practice is their application from peripheral blood to advance the diagnosis, improve prognosis, and monitor disease progression, instead of requiring more invasive procedures such as tissue biopsy or lumber punction. Despite the recent advancements in studying the potential role of EGMs as biomarkers for various diseases, the translation of these scientific findings into clinical practice has been limited, with notably progress primarily seen in the field of oncology. However, considering the findings of the aforementioned studies and the fact that EGMs can easily cross the BBB in various forms, including cfDNA, cf-mtDNA, noncoding RNAs, and EVs, EGMs hold considerable promise as valuable candidates for the psychiatric research.

Investigation of EGMs according to disease stages (such as acute exacerbation-remission or different stages of bipolar), disease subgroups, and phenotypic characteristics of patients holds the potential to uncover the related biological mechanisms and target more specific relevant pathways for treatment. Studies exploring the changes in expression levels of free EGMs, such as cimiRNAs and lncRNAs, as well as variations in metabolite and protein cargoes of EVs throughout different stages of the disease and in association with clinical phenotypes, would provide valuable insight into underlying mechanisms and pathways. Moreover, considering the overlapping symptomatology observed in different psychiatric diseases and the common genetic, neuroanatomical, and neurobiological findings, cross-disorders studies can also provide important information about at what points these diseases overlap and differ from each other, both biological and epigenetic wise. Furthermore, prospective studies with high-risk clinical individuals and prodromal patients may shed light on the biological processes associated with disease progression and transformation. Additionally, EGM studies have the potential to contribute valuable information on the neurobiological and epigenetic mechanisms underlying some environmental risk factors, such as stress, trauma and drug abuse, that contribute to the development of the diseases.

Another potential application of EGMs is to investigate the mechanisms of action of various treatment methods, such as lithium and electroconvulsive therapy (ECT), which are highly effective and widely used in treatment but still lack a complete understanding of underlying mechanisms. In this regard, EVs studies are very promising in particular. Analyzing changes in cargo contents following these treatments could help bridge the gap between neurobiology and clinical outcomes. For example, it has been hypothesized that ECT may act by inducing CNS cells to secrete EVs containing harmful and neurotoxic proteins/metabolites [124].

The research on CNS including post-mortem or preclinical studies which allow in situ analysis of the organ system has certain limitations, such as ethical concerns and criticism regarding to the extent to which these studies accurately reflect real diseases in vivo. However, the concept of analyzing the peripheral markers that are expected to reflect disease status in the CNS remains a subject of debate. Nevertheless, the potential of EGMs may overcome such obstacles. As previously mentioned, EGMs released from CNS cells can be detected peripherally, and their cell of origin can be distinguished using specific markers. These characteristics make them valuable tool for investigating the pathological processes occurring in the CNS in real time. Previous studies have investigated the involvement of EVs in transcription regulation, neurogenesis, synaptic plasticity, and neuroinflammation, all of which have been associated with psychiatric diseases [17]. Therefore, utilizing genomic and metabolomics/proteomic studies to examine the cargo content in CNS-derived EVs, along with the application of advanced technologies in the research field, will ultimately provide more precise information about the biological processes involved in the pathophysiology of psychiatric diseases.

Psychiatric disorders such as schizophrenia and bipolar disorder have some peripheral effects and are associated with comorbidities such as metabolic syndrome [128]. Given that EGMs play a role in cell-cell interaction and act as a kind of messenger between remote systems, EGM research may also help elucidating the underlying mechanisms of comorbid peripheral effects observed in psychiatric diseases. By qualitative and quantitative studies on EGMs, it may be possible to gain insight on the complex interplay between the central and peripheral systems in psychiatric diseases. This understanding could provide valuable information on the shared pathophysiology, potential biomarkers and therapeutic targets for both primary psychiatric disorders and its associated peripheral effects.

EGMs, particularly EVs, hold great promise for therapeutic advances in psychiatric disorders. EVs offer several advantages, including low immunogenicity and toxicity, biodegradability, the capacity to cross BBB while protecting their internal materials and the ability of being navigated to target cells and receptors. EV mediated drug delivery presents an exciting opportunity to develop more effective target-specific treatment approach while minimizing the systemic side effects [129]. Studies have already demonstrated the therapeutic potential of EVs derived from human mesenchymal stem cells in preclinical models of psychiatric disorders. These EVs have been shown to improve cognitive skills, enhance social interactions, and attenuate schizophrenia like behaviors by promoting the survival of parvalbumine positive GABA-ergic neurons and modulating neurotransmitter activity in CNS [130]. Recent advances in nanotechnology and genetic engineering allow for the manipulation of nucleic acid, protein and metabolite cargoes within EVs, tailoring them to the specific needs of target cells. Through engineering strategies, specific ligands involved in receptor mediated cellular communication can be enriched on the surface of EVs, enabling precise modulation of downstream pathways with inhibitory or excitatory effects. Thus, genetically or chemically engineered EVs may take treatment of psychiatric disorders one step further by allowing personalized and biological mechanism-based treatment options. Considering that nucleic acids encapsulated in EVs are protected from degradation by ribonucleases, even gene engineering through established ligands and gene therapy with exogenous genetic materials, such as miRNAs in EVs, would be possible with the advances in genomics of psychiatric disorders. In addition to potential benefits of EVs for therapeutic purposes, another therapeutic target associated with EGMs might be to neutralize proinflammatory effects of cfDNA by nucleic acid binding polymers in order to control abnormal immune response observed in psychiatric patients.

Overall, the use of EGMs presents exciting opportunities for advancing therapeutic interventions in psychiatric disorders, ranging from targeted drug delivery to gene therapy and immune modulation. Continued research in this field holds promise for developing innovative and personalized treatment strategies that address the complex pathophysiology of psychiatric disorders. Furthermore, it is widely acknowledged that relying on a single biomarker may not suffice to attain the desired level of specificity and sensitivity necessary for clinical application. Hence, although EGMs hold great promise for unraveling the mechanisms underlying psychiatric disorders, the translation of ongoing endeavors to identify molecular biomarkers for disease diagnosis and management necessitates further collaborative efforts from the research and medical communities. We hope that this review will provide a comprehensive foundation for future investigations and will serve as a guiding resource for researchers in this field.

## Materials and methods

PubMed was searched for all published articles through March 31st 2023, to include all articles examining any type of EGMs in patients with psychiatric disorders in comparison to healthy controls. The search was limited to English-written articles. The following search strategy were used: psychiatry* OR psychosis OR psychotic OR schizophrenia OR depression OR bipolar OR mania OR manic OR autism OR autistic OR Asperger OR anxiety OR ocd OR “obsessive compulsive” OR panic AND exosome OR microparticle OR virtosome OR nucleosome OR polynucleosome OR “cell-free DNA" OR cfDNA OR "cell-free DNA" OR "circulating DNA"OR "mitochondrial DNA" OR mtDNA OR "circular DNA"OR "cell-free RNA" OR "cell-free RNA" OR OR "extracellular trap*" OR "neutrophil-extracellular" OR “extracellular RNA" OR siRNA OR "circular RNA" OR miRNA OR microvesicles*

After merging and curating our results, we found 78 studies that met inclusion criteria: 22 for schizophrenia spectrum disorder, 10 for bipolar disorder, 22 for depressive disorders, 15 for autism spectrum disorder and 9 for other psychiatric disorders. Review articles were manually searched for any additional relevant studies.
