# Correction: The role of Extracellular Genomic Materials (EGMs) in psychiatric disorders

**DOI:** 10.1038/s41398-023-02589-x

**Published:** 2023-08-28

**Authors:** Ayşe Kurtulmuş, Cemal Çağıl Koçana, Selin Fulya Toprak, Selçuk Sözer

**Affiliations:** 1 Department of Genetics, Aziz Sancar Institute of Experimental Medicine, Istanbul University, Istanbul, Turkey; 2 Institute of Health Sciences, Istanbul University, Istanbul, Turkey; 3 Istanbul Göztepe Prof.Dr.Süleyman Yalçın City Hospital, Department of Psychiatry, Istanbul, Turkey

**Keywords:** Personalized medicine, Clinical genetics

Correction to: *Translational Psychiatry* 10.1038/s41398-023-02549-5, published online 18 July 2023

The Funding information section was missing from this article and should have read: The present work was supported by the Research Fund of Istanbul University. Project No. TYL-2019-33725.

The original article has been corrected.

